# Delayed Abscopal Response 3 Years After Robotic Stereotactic Body Radiation Therapy for Renal Cell Carcinoma: A Case Report

**DOI:** 10.1002/cnr2.70229

**Published:** 2025-05-08

**Authors:** Zhe Chen, Toshihiro Suzuki, Zennosuke Mochizuki, Hiroshi Takahashi, Kan Marino, Takafumi Komiyama, Hiroshi Onishi

**Affiliations:** ^1^ Department of Radiology University of Yamanashi Hospital Chuo Yamanashi Japan; ^2^ CyberKnife Center Kasugai General Rehabilitation Hospital Fuefuki Yamanashi Japan

**Keywords:** abscopal effect, CyberKnife, renal cell carcinoma, stereotactic body radiation therapy

## Abstract

**Background:**

Renal cell carcinoma (RCC) is the most common malignant tumor of the kidney in adults, with poor prognosis in advanced or metastatic stages. Although traditionally considered radioresistant, RCC has shown a promising response to stereotactic body radiation therapy (SBRT), which not only offers local tumor control but may also induce an abscopal effect, resulting in regression of distant metastases.

**Case:**

A 54‐year‐old male with a history of right RCC underwent radical nephrectomy in 2000, followed by partial nephrectomy for a left kidney recurrence in 2005. In 2011, imaging revealed a second recurrence in the left kidney. In 2013, after declining further surgery, he was treated with SBRT for the recurrent left renal lesion. Follow‐up imaging revealed a stable renal mass and a solid right lung nodule. Retrospective analysis of prior imaging suggested the presence of pulmonary metastases concurrent with the renal recurrence. The pulmonary nodule progressively enlarged until November 2016 but then spontaneously regressed by November 2017 without any additional systemic or local interventions, and remained stably reduced in size thereafter. Serial imaging from 2018 showed no evidence of new metastatic disease, and the left renal lesion exhibited partial regression. The pulmonary metastasis remained stable, consistent with the occurrence of an abscopal effect that persisted for 3 years. The patient ultimately passed away in 2024 from unrelated causes.

**Conclusion:**

This case demonstrates the potential of SBRT to induce a systemic abscopal response in metastatic RCC, with sustained control of pulmonary metastasis over 3 years. The findings suggest that SBRT may play a critical role in managing metastatic RCC, warranting further research into its synergy with immunotherapy for long‐term therapeutic benefit.

## 1 Introduction

Renal cell carcinoma (RCC) is the most common kidney tumor in adults and accounts for approximately 2%–3% of all cancers worldwide [1]. In advanced stages or cases involving metastasis, RCC is often associated with a poor prognosis. The initial management of RCC typically involves nephrectomy or partial nephrectomy, which aims to achieve curative outcomes for localized disease. However, recurrence and metastasis frequently necessitate additional interventions, including systemic therapies or further surgery [2].

Historically, radiation therapy (RT) was considered to have limited efficacy for RCC due to its intrinsic radioresistance [3]. This resistance was attributed to RCC's slow cell cycle and efficient DNA repair mechanisms, which reduced the cytotoxic effects of RT. Nonetheless, technological advances, particularly in the form of stereotactic body radiation therapy (SBRT), have challenged this notion. SBRT, which delivers high‐dose, precise radiation to tumors while sparing surrounding tissues, has emerged as a promising option for RCC treatment [4].

In addition to local tumor control, SBRT has been associated with the “abscopal effect.” This rare phenomenon involves the regression of non‐irradiated metastatic lesions, presumably through radiation‐induced immune activation. The current case provides compelling evidence for the abscopal effect in RCC, observed through the spontaneous regression of lung metastases following CyberKnife SBRT for a renal tumor. This highlights the need to further investigate into RT's systemic effects and their implications for RCC management.

## 2 Case Presentation

**FIGURE 1:**
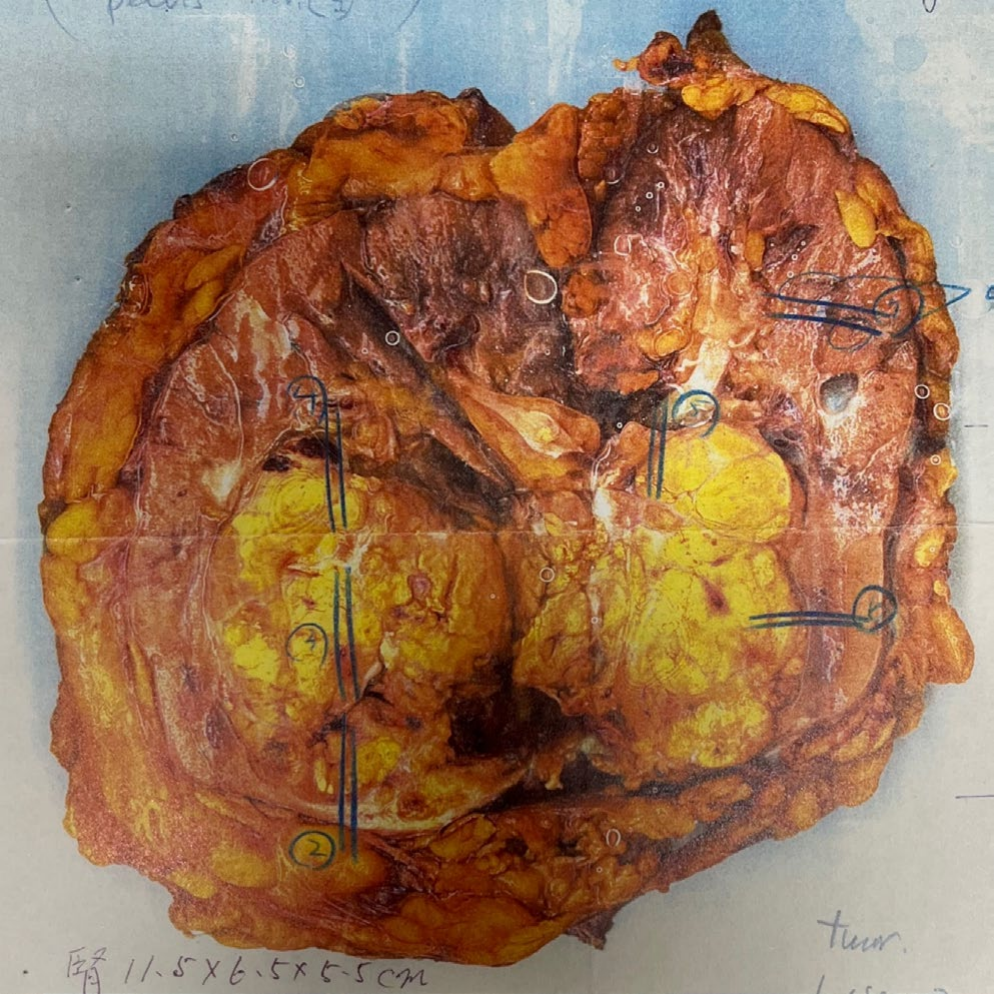
Gross pathology of the resected right kidney. The specimen weighed 350 g and measured 11.5 × 6.5 × 5.5 cm. A well‐circumscribed, yellowish, partially hemorrhagic mass (6.0 × 4.0 × 3.0 cm) was located in the lower two‐thirds of the kidney and classified as pathological stage pT1b.

A 54‐year‐old male was initially diagnosed with right RCC in October 2000 and underwent radical nephrectomy at an outside institution. The resected right kidney (Figure 1) weighed 350 g and measured 11.5 × 6.5 × 5.5 cm. Gross pathological assessment revealed a well‐demarcated, yellowish, partially hemorrhagic mass measuring 6.0 × 4.0 × 3.0 cm, located in the lower two‐thirds of the kidney and classified as pT1b. Microscopic evaluation confirmed clear cell RCC, grade 2, with interferon‐α positivity and pV0 staging. Necrosis, hemorrhage, and calcification were infrequently observed in the tumor tissue. The ureteral margin was negative for malignancy, and no metastatic involvement was identified in the right adrenal gland. The postoperative course was uneventful, and the patient remained recurrence‐free until June 2005, when a new lesion was identified in the contralateral (left) kidney. Partial nephrectomy was performed, achieving temporary disease control. Histopathological findings were consistent with metastatic clear cell RCC, originating from the right kidney.

**FIGURE 2:**
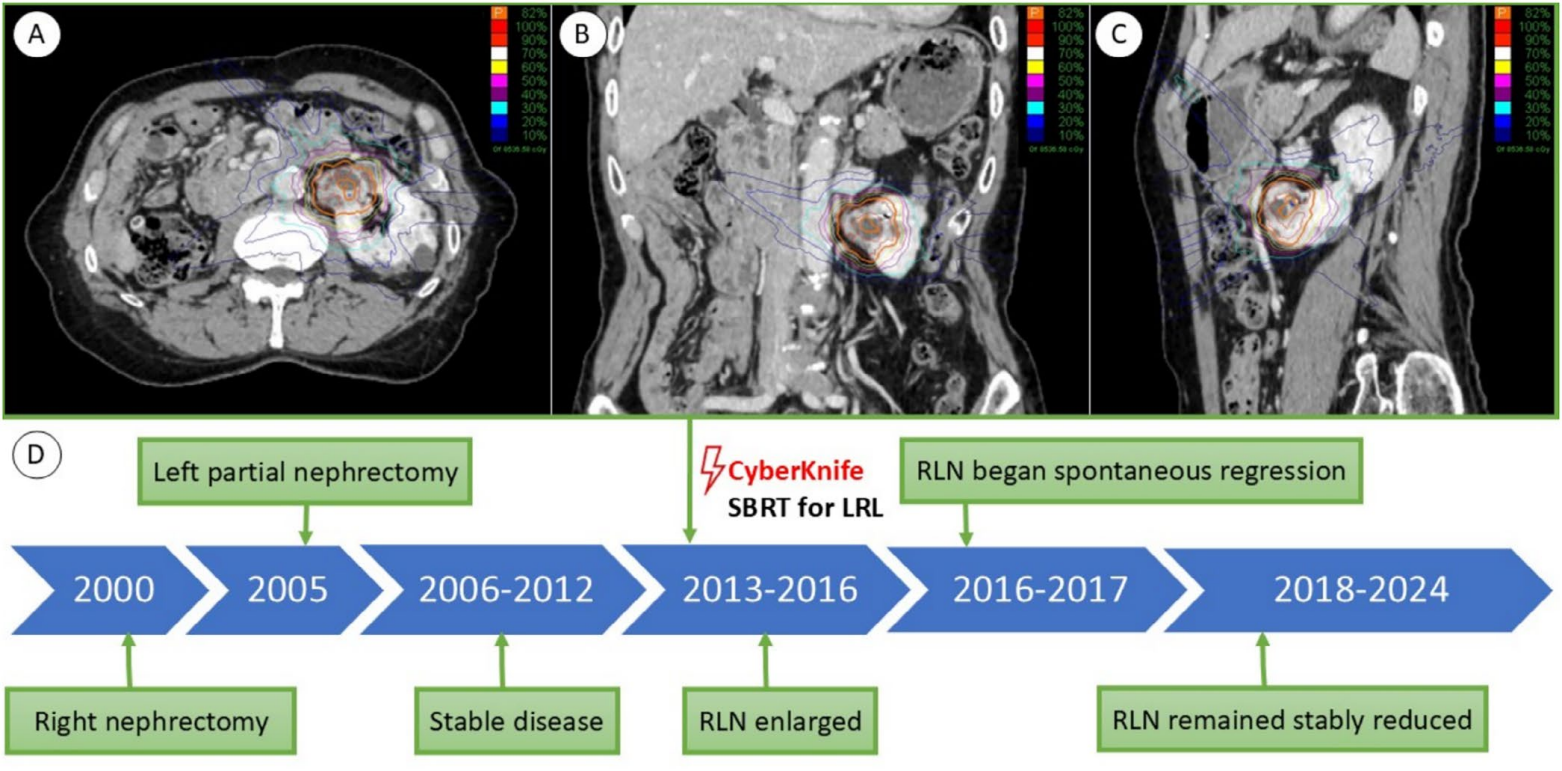
CyberKnife treatment planning and clinical timeline. Panels (A)–(C) display the dose distribution for stereotactic body radiation therapy (SBRT) to the left renal lesion in axial (A), coronal (B), and sagittal (C) views. Panel (D) illustrates the clinical timeline: Right nephrectomy in 2000, left partial nephrectomy in 2005, and CyberKnife SBRT in 2013. A right lung nodule (RLN), retrospectively visible in 2013, enlarged until 2016 and spontaneously regressed by late 2017, remaining stably reduced in size thereafter. LRL, left renal lesion; RLN, right lung nodule; SBRT, stereotactic body radiation therapy.

In March 2011, imaging revealed a second recurrence in the left kidney. Given the tumor's deep parenchymal location, radical nephrectomy was advised; however, the patient declined surgery due to his desire to avoid dialysis. He actively sought non‐surgical therapeutic alternatives and was referred in March 2013 to our CyberKnife center (Kasugai Rehabilitation Hospital, Fuefuki, Japan) for consideration of SBRT. In April 2013, the patient underwent SBRT using the CyberKnife system, receiving a total dose of 70 Gy in 10 daily fractions (Figure 2A–C). A detailed timeline of treatment and follow‐up is illustrated in Figure 2D. Follow‐up computed tomography (CT) in October 2013 demonstrated a stable residual lesion in the left kidney, along with a solid, cannonball‐like nodule in the right lung. Retrospective review of earlier imaging identified a subtle ground‐glass nodule in the same location on a CT scan obtained in March 2013, suggesting that pulmonary metastases had likely been present concurrently with the renal recurrence. The pulmonary nodule gradually enlarged, reaching its maximal diameter by November 2016, but subsequently underwent spontaneous regression by November 2017 in the absence of any additional systemic or local therapy and remained stably reduced in size thereafter. The irradiated left renal lesion showed only modest size reduction over time, while renal function exhibited progressive decline.

**FIGURE 3:**
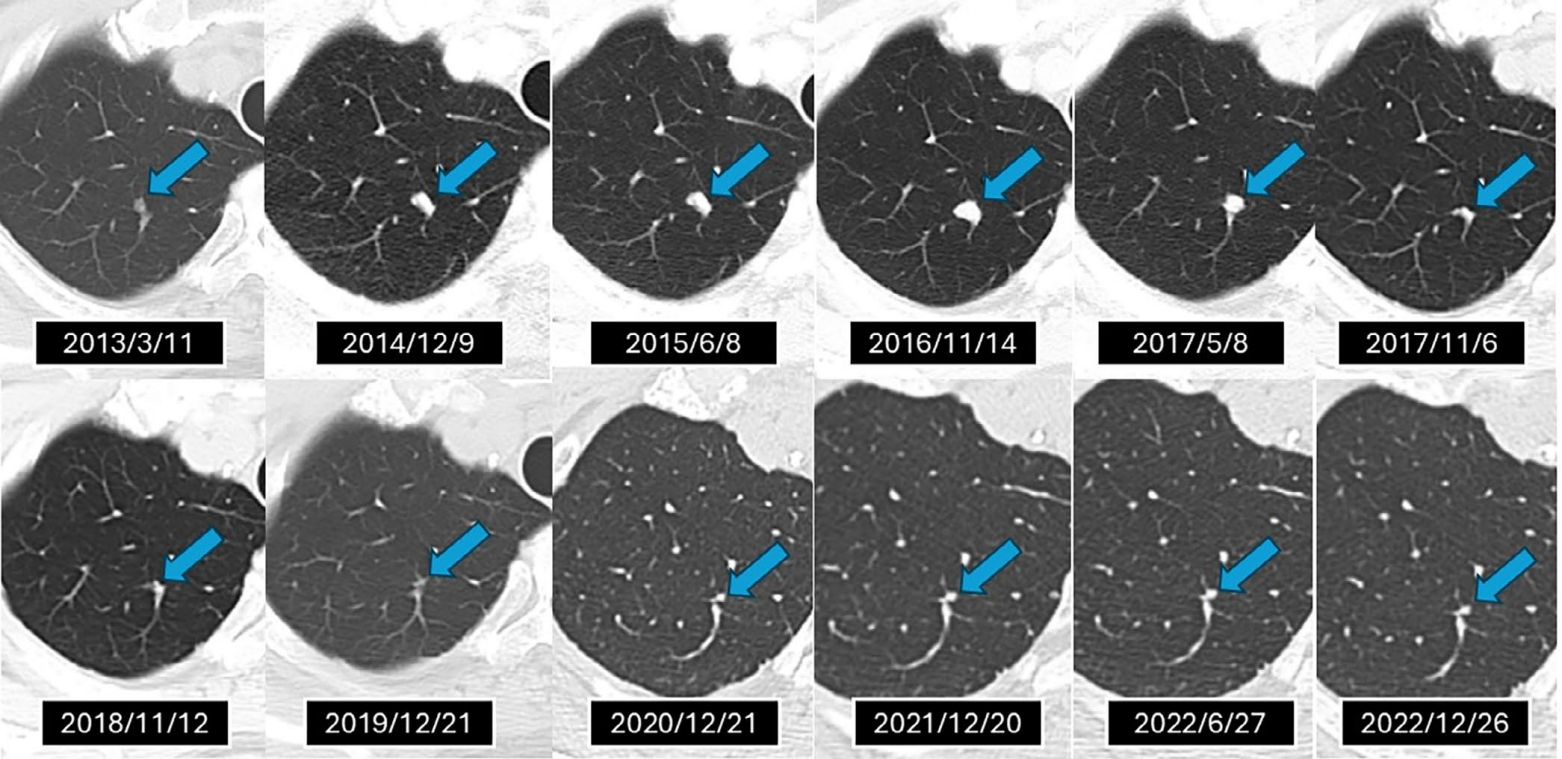
Radiographic course of the pulmonary metastasis demonstrating an abscopal response following stereotactic body radiation therapy (SBRT) with the CyberKnife system. A small ground‐glass nodule in the right lung was retrospectively identified on a CT scan from March 2013, prior to SBRT to the left renal lesion in April 2013. The nodule gradually enlarged, peaking in size by November 2016, then spontaneously regressed without further treatment and remained stably reduced in size thereafter. Serial imaging through December 2022 confirmed its sustained size reduction, with no CT scans performed thereafter.

From November 2018 onward, serial imaging demonstrated no evidence of new metastatic disease. The left renal mass remained morphologically stable, and the right lung lesion remained stably reduced in size on imaging, with no evidence of regrowth. By June 2023, a partial response of the irradiated renal lesion was noted, with continued stability of the pulmonary metastasis (Figure 3). The pulmonary metastasis began to regress spontaneously within 1 year following its maximum size in November 2016 and remained radiographically stable thereafter. The patient ultimately passed away in October 2024 due to causes unrelated to RCC.

## 3 Discussion

This case illustrates a rare and durable instance of the abscopal effect following SBRT for RCC, a traditionally radioresistant tumor. While RT has not been a standard component of RCC management, this case demonstrates that SBRT can achieve systemic anti‐tumor effects beyond local control, challenging the conventional treatment paradigms.

### 3.1 Clinical Evidence of the Abscopal Effect in RCC

Although reports of the abscopal effect in RCC are rare, similar cases have been documented. Fairlamb et al. [5] described a 73‐year‐old woman with metastatic RCC who experienced regression of pulmonary metastases following palliative RT. Similarly, MacManus et al. [6] observed lung metastasis regression in a 58‐year‐old man after low‐dose RT, with evidence of immune activation via elevated interleukin‐2 receptor levels. Other notable cases include Ishiyama et al. [7], who reported systemic regression in a patient with widely metastatic RCC following SBRT, and Wersäll et al. [8], who observed abscopal effects in four RCC cases treated with SBRT. These studies highlight SBRT's potential to induce immune‐mediated tumor regression, with some reporting higher rates of abscopal effects compared to spontaneous regression in RCC.

While most documented abscopal effects occur within 2 months of RT [9], this case is unusual for the delayed onset and prolonged persistence of the response. The pulmonary metastasis began to regress approximately 3 years after SBRT and showed sustained radiographic stability. This represents one of the longest observed durations of an abscopal effect in RCC, providing compelling evidence of SBRT's lasting systemic impact in a traditionally radioresistant tumor.

### 3.2 Radiological Assessment of Pulmonary Metastases

The lungs represent the most frequent site of metastasis in RCC, with pulmonary involvement occurring in approximately 50%–60% of cases with distant spread [10]. On imaging, pulmonary metastases typically manifest as well‐defined, round nodules—commonly referred to as “cannonball” lesions—ranging from 0.5 to 2 cm in diameter. These lesions are often asymptomatic and are frequently detected incidentally during routine follow‐up [10].

In the present case, a solitary pulmonary nodule with a characteristic cannonball appearance was detected on contrast‐enhanced CT. Based on its imaging features and distribution, the lesion was highly suggestive of metastatic RCC. Notably, throughout the observation period, the patient exhibited no clinical signs of pulmonary infection, nor were there radiological findings suggestive of pneumonia in the lung parenchyma surrounding the nodule. Furthermore, the entire course—from initial detection of the nodule, through its gradual enlargement, to spontaneous resolution—spanned a period of 4 years, a temporal pattern that is not consistent with a benign inflammatory response.

Histopathologic confirmation was not pursued due to the small size of the lesion and the patient's preference to avoid invasive procedures. The diagnosis was instead supported by expert consensus from radiologists specializing in oncologic imaging.

Notably, the solitary pulmonary lesion demonstrated regression following SBRT to the primary renal tumor. Although the response was delayed, the temporal association supports the possibility of an abscopal effect, further reinforcing the metastatic nature of the lung lesion. Contrast‐enhanced CT is the preferred imaging modality for the detection and assessment of pulmonary metastases due to its high sensitivity, particularly for small nodules [11]. In contrast, FDG‐PET has limited sensitivity in this setting (approximately 63%), especially for lesions smaller than 2 cm, and is not routinely recommended [12].

With the progressive increase in the average age of patients with RCC and the associated decline in overall physical status, reliance on imaging‐based diagnosis for identifying metastatic lesions is anticipated to become increasingly common [13]. RCC is also characterized by its indolent behavior, and tumor regression, when observed, often occurs over a prolonged period [14, 15]. Thus, the minimal change in the primary tumor is consistent with its known biological behavior. The earlier regression of the metastatic lung lesion may indeed reflect an immune response, possibly triggered by the SBRT.

### 3.3 Mechanisms of the Abscopal Effect in RCC

The abscopal effect refers to the regression of non‐irradiated tumors following localized radiation and is thought to occur via activation of the immune system [16]. RT‐induced DNA damage releases tumor antigens and cytokines, activating T‐cells that target distant tumors [9]. Additionally, antigen‐presenting cells can capture these tumor‐derived antigens and migrate to lymph nodes, where they prime T cells against tumor‐specific antigens. Activated T cells, primed against tumor‐specific antigens, can then infiltrate both the irradiated primary tumor and distant, non‐irradiated metastases, mediating an abscopal effect [17]. RT may also shift the tumor microenvironment from immunosuppressive to immunostimulatory, further enhancing systemic responses.

### 3.4 Hypothesized Mechanism for Delayed Onset

The delayed onset of the abscopal effect observed in our case is atypical and warrants further discussion. Given the known mechanism of T cell activation through radiation‐induced antigen release, the timing of the immune response may be influenced by tumor biology. RCC is characterized by slow progression [18], and tumor regression can unfold over several years [14, 19]. We therefore hypothesize that the delayed abscopal effect in this case reflects a prolonged release of tumor‐associated antigens, gradually priming the immune system over time. In this context, late activation of T cells could account for the regression of the pulmonary metastasis observed more than 4 years after SBRT.

### 3.5 Differentiating Abscopal Effect From Spontaneous Regression

The abscopal effect has historically been classified as a form of spontaneous regression, a term initially used to describe phenomena that were poorly understood. However, spontaneous regression does not imply that these effects arise independently of physical or biological triggers. Recent evidence suggests that ionizing radiation, particularly at higher doses, can induce immunomodulatory effects through inflammatory responses, providing a plausible mechanism for the abscopal effect [20].

Additionally, cases of abscopal effects occurring years after RT have been documented. For example, Wersäll et al. [8] reported abscopal responses 2 years post‐RT. In Table 1, we summarize all reported cases of abscopal effects in metastatic RCC to date, highlighting the diversity in timing and clinical presentation. This case highlights the diversity in the timing and presentation of abscopal effects, challenging the notion that such responses are limited to the subacute period following radiation. It also raises the question of how many cases historically classified as spontaneous regression might actually represent abscopal effects triggered by prior interventions.

**TABLE 1:** Summary of reported cases of abscopal effects in metastatic renal cell carcinoma.

No.	Author	Age	Gender	Total dose (Gy)	Fractions	RT technique	Site treated with RT	Site with AE	Time for the AE to appear (months)	Duration of AE (months)	Combined with ICIs	Follow‐up duration (months)
1	Fairlamb [5]	73	F	40	15	2D	Pubic bone	Lung	2	NA	NA	54
2	Macmanus [6]	58	M	20	10	NA	Renal mass	Lung and mediastinum	6	NA	NA	11
3	Wersäll [8]	83	F	32	4	SBRT	Renal mass	Lymph node and lungs	24	NA	NA	NA
4	Wersäll [8]	64	F	NA	NA	SBRT	Lung lesions	Lung	NA	NA	NA	48
5	Wersäll [8]	69	M	NA	NA	SBRT	Lung lesions	Lung	NA	NA	NA	24
6	Wersäll [8]	55	F	32	4	SBRT	Renal mass	Lung	5	NA	NA	9
7	Ishiyama [7]	61	M	40	5	SBRT	Bone and spine	Lung	1	NA	NA	36
8	Present study	54	M	70	10	SBRT	Renal mass	Lung	43	55	N	138

Abbreviations: AE, abscopal effect; ICI, immune checkpoint inhibitor; NA, not available; No., number; RT, radiation therapy; SBRT, stereotactic body radiation therapy.

Emerging evidence suggests that combining RT with immune checkpoint inhibitors (ICIs) or tyrosine kinase inhibitors can enhance the abscopal effect by accelerating immune activation [17, 21]. In the present case, the absence of such therapies underscores the potential of SBRT as an effective monotherapy. Exploring its combination with immunotherapy may further optimize outcomes, particularly in metastatic RCC. Notably, the prolonged responses in this case occurred without concurrent immunotherapy, which is uncommon, as most long‐lasting abscopal effects are observed in conjunction with ICIs [22]. These findings underscore the capacity of CyberKnife SBRT alone to induce durable systemic effects.

Future research should focus on identifying biomarkers predictive of abscopal responses, as well as determining the most effective radiation doses and schedules [23]. Such studies could refine RCC management and expand the therapeutic potential of SBRT for traditionally radioresistant tumors.

## 4 Limitations

This case has several limitations. First, as the surgeries were performed 20 and 25 years ago at other hospitals, only partial pathological records were available despite our efforts, and complete records could not be obtained. Second, the diagnosis of pulmonary metastases was based solely on imaging without pathological confirmation. Third, distinguishing the abscopal effect from spontaneous regression—a phenomenon more common after primary tumor removal—remains challenging. Although the timing and incidence of the abscopal effect are not well defined, and its occurrence during the subacute phase of RT is not always certain, it is important to note that the abscopal effect was historically grouped under spontaneous regression, as such phenomena were initially considered unexplained. These complexities underscore the need for further research to better understand these mechanisms.

## 5 Conclusion

This case report highlights the potential of SBRT to induce systemic effects, such as the abscopal effect, in metastatic RCC, underscoring its role beyond local tumor control. The peculiarity of this case lies in the sustained abscopal response observed over a prolonged period, which is rare in RCC and provides valuable insights into the systemic impact of radiotherapy. Importantly, this case demonstrates that in patients with multiple metastatic lesions, targeting the largest or symptomatic lesion while closely monitoring others may be a viable strategy, as the abscopal effect could offer additional therapeutic benefits. These findings suggest the need for further research to explore the combination of SBRT with immunotherapy and its long‐term implications for metastatic RCC management.

## Author Contributions

Z.C. contributed to conceptualization, data curation, and wrote the original draft. T.S. and Z.M. performed data curation and investigation. H.T., K.M., and T.K. critically revised the manuscript. H.O. supervised the study and participated in manuscript revision.

## Ethics Statement

This retrospective case report analysis used fully de‐identified data, qualifying for IRB exemption per our institutional ethics policy. A statement of written informed consent was obtained from the deceased patient's next of kin (spouse) for publication of this case report, including all clinical details and medical images.

## Conflicts of Interest

The authors declare no conflicts of interest.

## Data Availability

The authors have nothing to report.
